# Fibrinogen-to-albumin ratio (FAR) is the best biomarker for the overall survival of patients with non-small-cell lung cancer

**DOI:** 10.3389/fonc.2024.1396843

**Published:** 2024-06-24

**Authors:** Shixin Ma, Lunqing Wang

**Affiliations:** ^1^ Graduate School, Dalian Medical University, Dalian, Liaoning, China; ^2^ Department of Thoracic Surgery, Qingdao Municipal Hospital, Qingdao, Shandong, China

**Keywords:** non-small cell lung cancer, inflammatory index, nutritional index, prognosis, overall survival

## Abstract

**Objective:**

The inflammatory response and the nutritional status are associated with overall survival (OS) in patients with non-small cell lung cancer (NSCLC), but it is unclear which biomarkers are better suited to predict prognosis. This study sought to determine which of the commonly existing inflammatory and nutritional indicators best predicted the OS.

**Methods:**

This study included 15 compound indicators based on inflammation or nutrition, with cutoff points obtained through the receiver operating characteristic (ROC) curve. Univariate and multivariate Cox proportional risk models were used to evaluate the relationship between these predictors and OS. Kaplan–Meier curves were used for survival analysis, and log-rank tests were used to compare differences between groups. The C-index was calculated to evaluate the predictive ability of the different indicators.

**Results:**

The study included 899 patients with NSCLC. In the univariate analysis, all 15 measures were significantly associated with the OS of patients (all *p* < 0.05). The results of the C-index analysis showed that the fibrinogen-to-albumin ratio (FAR), the systemic immune-inflammation index (SII), and the albumin-to-alkaline phosphatase ratio (AAPR) were the three indices with the best predictive performance. Among them, FAR (C-index = 0.639) had the best predictive power for OS in patients with NSCLC. In the different subgroups, FAR had the highest C-index in male, non-smoking, adenocarcinoma, and stage II patients. The C-index of the platelet-to-lymphocyte ratio (PLR) in female patients was the highest. SII was the highest in smokers, in those aged <65 and ≥65 years, and in stage III patients. The C-index of AAPR was the highest in non-adenocarcinomas. The C-index of the pan-immune-inflammation value (PIV) was the highest in stage I patients. In the multivariate Cox regression analysis, among FAR, SII, and AAPR, only FAR was an independent predictor of OS in patients with NSCLC. A high FAR was associated with a higher risk of death in patients with NSCLC (HR = 1.601, 95% CI = 1.028–2.495). In order to further evaluate the potential prognostic value of FAR, SII, and AAPR in patients with different stages, Cox regression analysis was performed for those with stage I–II and stage III NSCLC. The results showed that FAR was an independent prognostic factor for OS in patients with stage I–II NSCLC.

**Conclusion:**

For all patients with NSCLC, the prognostic power of FAR was superior to that of other inflammatory and nutritional indicators.

## Background

Lung cancer represents 11.4% of all malignancies and causes 18% of all cancer-related deaths, making it the primary cause of cancer mortality according to the Global Cancer Statistics 2020 report (1). Among these, non-small cell lung cancer (NSCLC) accounts for approximately 80%–85% of all lung cancer cases and is associated with a poor 5-year survival rate (2). Patients with early-stage NSCLC are mainly treated with surgery, and postoperative adjuvant therapy is usually recommended thereafter to prevent cancer recurrence and to effectively improve patient survival (3). With significant advances in clinical diagnosis and treatment techniques and in antitumor treatment options (such as immunotherapy), 5-year survival rates have improved (4). However, predicting the prognosis of patients with lung cancer is still challenging. Therefore, there is an urgent need for effective biomarkers to predict the survival prognosis of patients in order to help identify patients and conduct timely and effective treatment. The American Joint Committee on Cancer (AJCC) tumor node metastasis staging system is an important factor in the assessment of the prognosis of patients with NSCLC (5). In addition, the patient’s age, sex, smoking status, weight status, and other clinical indicators are also factors that affect the survival outcomes of individual cancer patients (6–8).

Recent research has indicated that many inflammation and nutritional markers can serve as reliable prognostic indicators for lung cancer. The indicators of systemic inflammatory response, including the neutrophil-to-lymphocyte ratio (NLR) (9), the platelet-to-lymphocyte ratio (PLR) (10), the lymphocyte-to-monocyte ratio (LMR) (11), the systemic immune-inflammation index (SII) (12), and the pan-immune-inflammation value (PIV) (13), are significant in determining the prognosis of patients with lung cancer. The fibrinogen-to-albumin ratio (FAR) (14), the fibrinogen-to-prealbumin ratio (FPR) (15), the albumin-to-globulin ratio (AGR) (16), the advanced lung cancer inflammation index (ALI) (17), the prognostic nutritional index (PNI) (18), the albumin-to-alkaline phosphatase ratio (AAPR) (19), and the C-reactive protein-to-albumin ratio (CAR) (20), as well as other inflammatory and nutritional complex indicators, are also biomarkers for predicting the prognosis of lung cancer. Furthermore, red cell distribution width-to-albumin ratio (RAR) (21), alkaline phosphatase-to-prealbumin ratio (APR) (22), free fatty acid (FFA) content (23), and other markers are significant predictors for cancer patients. However, despite the predictive value of RAR, APR, and FFA in cancer, their prognostic value for lung cancer remains unknown.

Although studies have confirmed the value of some of the aforementioned nutrition- and inflammation-related indicators in predicting the survival outcomes of patients with lung cancer, it is necessary to identify which indicators are the best prognostic factors in patients with NSCLC. In this study, we evaluated and compared the predictive value of 15 nutritional and inflammatory biomarkers for overall survival (OS) for patients with NSCLC. We also assessed which indicator had a higher predictive value in different groups.

## Materials and methods

### Object of study

**Figure 1:**
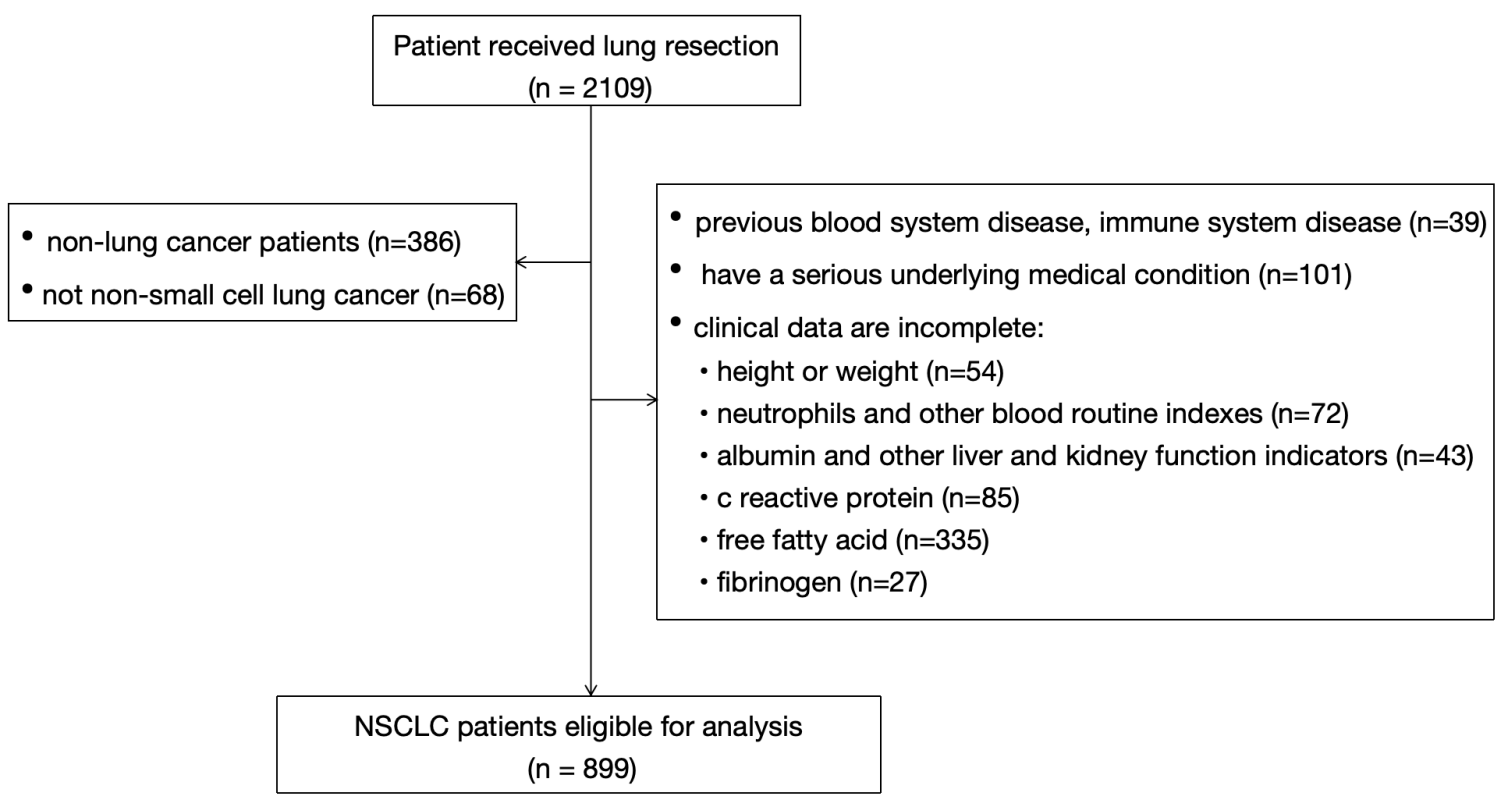
Patient screening flowchart.

Patients with NSCLC who received surgical treatment in our hospital from January 2017 to June 2021 were selected as the study subjects. All of the patients included in this study met the following criteria: 1) at least 18 years of age; 2) undergoing thoracoscopic surgery for pathologically proven NSCLC (classified as stage I–III lung cancer according to TNM edition 8) (24); 3) no previous history of malignant tumors or the presence of a second primary cancer; and 4) with complete preoperative clinical data available. The exclusion criteria were as follows: 1) patients with NSCLC that is unresectable or who cannot tolerate surgical treatment; 2) patients with blood system and immune system diseases or blood abnormalities of unknown cause; 3) those with severe underlying disease in the past (such as grade IV heart function, liver and kidney failure, and stroke with severe sequelae, among others), resulting in unclear outcome indicators; and 4) those with incomplete clinical data or incomplete follow-up records. The flowchart for patient screening is shown in **Figure 1**.

### Data collection

In this study, the baseline information of the patients, including age, sex, smoking status, and body mass index (BMI), was collected through the hospital’s electronic medical record filing system, quality control registration management system, and laboratory examination reporting system. The Eastern Cooperative Oncology Group Performance Status (ECOG PS) score, respiratory diseases, tumor diameter, pathological type and TNM stage, the presence of vascular invasion, endovascular thrombus, lymphatic vessel invasion, and perineural invasion, and the survival status of the patients, among others, were assessed.

### Measurement of nutritional/inflammatory markers

After fasting for at least 9 h, routine blood tests were performed within 24 h of hospitalization to measure the inflammatory indicators (including neutrophils, lymphocytes, monocytes, C-reactive protein, platelets, serum FFAs, globulins, alkaline phosphatase, and fibrinogen) and the nutritional status indicators (including albumin, prealbumin, and red blood cell distribution width). The 15 nutrition/inflammation-based measures used in this study included FPR, CAR, AAPR, FAR, APR, RAR, AGR, NLR, LMR, FFA/Alb, PNI, ALI, PLR, PIV, and SII. The methodology for the calculation of each nutrition/inflammation indicator and the specificity and sensitivity of the receiver operating characteristic (ROC) curve can be found in **Supplementary Tables S1**, **S2**, respectively.

### Follow-up

The patients were followed up according to the hospital’s outpatient and inpatient medical record system and by telephone, with the follow-up ending in December 2022. The date when the patients were pathologically diagnosed with NSCLC was first defined as the starting point, death due to lung cancer was defined as the outcome event, and the Overall Survival (OS) was the time from the starting point to death or the end of follow-up.

### Statistical methods

The SPSS 27.0 software, R4.2.1 software, and GraphPad Prism 9.4.1 software were used for data processing and analysis. The measurement data were expressed as the mean plus or minus the standard deviation or median (quartile range), while the counting data were expressed as the number of cases (percentage). Differences between groups were assessed using the Mann–Whitney *U* test (for continuous variables with non-normal distributions) and the chi-squared test (for categorical variables). The best cutoff values for each inflammatory or nutritional index were obtained using the ROC curve and were divided into two categorical variables. Survival analysis was presented by the Kaplan–Meier curve, and differences between groups were compared using the log-rank test. Cox’s proportional hazards regression models were used to examine the relationship between nutritional and inflammatory markers and OS in patients with NSCLC. The predictive accuracy of each indicator was assessed using the C-index. To determine whether the same indicator applied to the entire subgroup and to gain insight into the most valuable biomarkers in the different subgroups, subgroup analyses were performed for patients’ age, sex, pathology type, and smoking status. A two-tailed *p*-value of <0.05 was considered statistically significant.

## Results

### Baseline characteristics

A total of 899 patients were included in the study. There were 413 men and 486 women. The mean age of the patients was 61.36 ± 9.64 years. Of all the patients in the study, 283 (31.5%) had a history of smoking, 220 (24.5%) had a history of lung disease, and 170 (18.9%) had an ECOG PS score of 2 or higher. In all patients, the mean tumor diameter was 1.96 ± 1.37 cm, 793 (88.2%) were pathologically diagnosed as adenocarcinomas, and 106 (11.8%) were non-adenocarcinomas. There were 707 (78.6%), 87 (9.7%), and 105 (11.7%) patients with TNM stages I, II, and III, respectively. Vascular invasion, endovascular thrombus, lymphatic vessel invasion, and perineural invasion were observed in 62.8%, 11.0%, 61.4%, and 2.7% of patients, respectively. In addition, the average BMI of the patients included in the study was 24.67 ± 3.38 kg/m^2^. The patient characteristics are shown in **Table 1**.

**Table 1:** Patient characteristics [*n* (%)].

Baseline	All (*n* = 899)
**Age, mean ± SD**	61.36 ± 9.64
**Gender, n (%)**	
Male	413 (45.9)
Female	486 (54.1)
**BMI, mean ± SD**	24.67 ± 3.38
**Smoking status, n (%)**	
Never smoking	616 (68.5)
Current or former smoker	283 (31.5)
**Respiratory diseases, n (%)**	
No	679 (75.5)
Yes	220 (24.5)
**ECOG PS score, n (%)**	
0 - 1	729 (81.1)
≥2	170 (18.9)
**Tumor diameter, mean ± SD**	1.96 ± 1.37
**Pathologic types, n (%)**	
Adenocarcinoma	793 (88.2)
Non-Adenocarcinoma	106 (11.8)
**TNM stage, n (%)**	
I	707 (78.6)
II	87 (9.7)
III	105 (11.7)
**Vascular invasion**	
No	334 (37.2)
Yes	565 (62.8)
**Endovascular thrombus**	
No	800 (89.0)
Yes	99 (11.0)
**lymphatic vessel invasion**	
No	347 (38.6)
Yes	552 (61.4)
**Perineural invasion**	
No	875 (97.3)
Yes	24 (2.7)
**FPR, median (IQR)**	0.011 (0.009 - 0.014)
**CAR, median (IQR)**	0.024 (0.012 - 0.083)
**AAPR, median (IQR)**	0.048 (0.040 - 0.059)
**FAR, median (IQR)**	0.076 (0.066 - 0.090)
**APR, median (IQR)**	0.30 (0.24 - 0.39)
**RAR, median (IQR)**	1.07 (0.98 - 1.17)
**AGR, median (IQR)**	1.44 (1.29 - 1.60)
**NLR, median (IQR)**	1.68 (1.28 - 2.28)
**LMR, median (IQR)**	4.37 (3.28 - 5.69)
**FFA/Alb, median (IQR)**	9.96 (6.94 - 11.59)
**PNI, median (IQR)**	49.42 (46.00 - 52.91)
**ALI, median (IQR)**	57.92 (41.83 - 79.26)
**PLR, median (IQR)**	119.55 (94.69 - 151.22)
**PIV, median (IQR)**	162.23 (108.91 - 275.94)
**SII, median (IQR)**	378.28 (270.21 - 546.24)

FPR, fibrinogen to prealbumin ratio; CAR, C-reactive protein to albumin ratio; AAPR, albumin to alkaline phosphatase ratio; FAR, fibrinogen to albumin ratio; APR, alkaline phosphatase to prealbumin ratio; RAR, red cell distribution width to albumin ratio; AGR, albumin globulin ratio; NLR, neutrophil to lymphocyte ratio; LMR, lymphocyte to monocyte ratio; FFA/Alb, Free fatty acid to albumin ratio; PNI, prognostic nutritional index; ALI, advanced lung cancer inflammatory index; PLR, platelet to lymphocyte ratio; PIV, Pan-Immune-Inflammation Value; SII, systemic immune inflammation index.

### Association of inflammatory and nutritional markers with OS in lung cancer patients

**Figure 2:**
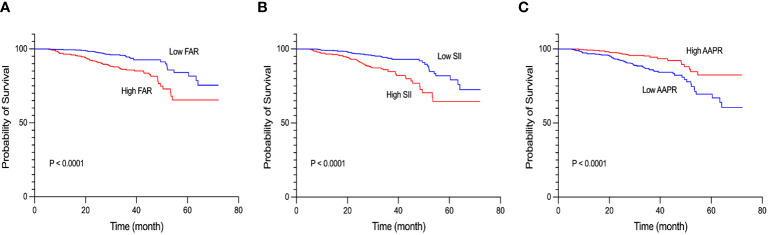
Kaplan–Meier curves of the fibrinogen-to-albumin ratio (FAR) **(A)**, systemic immune-inflammation index (SII) **(B)**, and albumin-to-alkaline phosphatase ratio (AAPR) **(C)** in patients with non-small cell lung cancer (NSCLC).

By December 2022, a total of 97 patients with lung cancer had died. The median OS was 34.23 months. The median OS for the adenocarcinoma and non-adenocarcinoma patients was 33.97 and 35.40 months, respectively. The cutoff points of the 15 inflammation- and malnutrition-based indicators were 0.013 (FPR), 0.017 (CAR), 0.045 (AAPR), 0.079 (FAR), 0.34 (APR), 1.13 (RAR), 1.49 (AGR), 1.64 (NLR), 4.17 (LMR), 9.81 (FFA/Alb), 45.51 (PNI), 70.06 (ALI), 143.73 (PLR), 156.18 (PIV), and 487.10 (SII). The univariate and multivariate analyses showed that FPR (*p* < 0.001), CAR (*p* = 0.001), AAPR (*p* < 0.001), FAR (*p* < 0.001), APR (*p* = 0.002), RAR (*p* < 0.001), AGR (*p* = 0.005), NLR (*p* < 0.001), LMR (*p* = 0.007), FFA/Alb (*p* = 0.005), PNI (*p* < 0.001), ALI (*p* < 0.001), PLR (*p* < 0.001), PIV (*p* < 0.001), and SII (*p* < 0.001) were independent risk factors affecting the survival of patients with NSCLC (**Table 2**). The Kaplan–Meier curves showed that lung cancer patients with malnutrition and inflammation have a more unfavorable OS compared with NSCLC patients without malnutrition or inflammation (**Figure 2**; **Supplementary Figure S1**).

**Table 2:** Univariate analysis of the 15 inflammatory/nutritional markers and overall survival (OS) in patients with non-small cell lung cancer.

Category	*n* (%)	Overall survival		
		HR	95%CI	*p*-value
FPR				
<0.013	627 (69.7)		1.000	
≥0.013	272 (30.3)	2.073	1.391–3.090	<0.001
CAR				
<0.017	386 (42.9)		1.000	
≥0.017	513 (57.1)	2.287	1.432–3.652	0.001
AAPR				
<0.045	368 (40.9)		1.000	
≥0.045	531 (59.1)	0.425	0.280–0.644	<0.001
FAR				
<0.079	527 (58.6)		1.000	
≥0.079	372 (41.4)	2.545	1.683–3.848	<0.001
APR				
<0.34	547 (60.8)		1.000	
≥0.34	352 (39.2)	1.878	1.256–2.809	0.002
RAR				
<1.13	606 (67.4)		1.000	
≥1.13	293 (32.6)	1.713	1.147–2.559	<0.001
AGR				
<1.49	528 (58.7)		1.000	
≥1.49	371 (41.3)	0.527	0.337–0.825	0.005
NLR				
<1.64	424 (47.2)		1.000	
≥1.64	475 (52.8)	2.529	1.604–3.987	<0.001
LMR				
<4.17	408 (45.4)		1.000	
≥4.17	491 (54.6)	0.572	0.383–0.856	0.007
FFA/Alb				
<9.81	431 (47.9)		1.000	
≥9.81	468 (52.1)	0.541	0.351–0.833	0.005
PNI				
<45.51	198 (22.0)		1.000	
≥45.51	701 (78.0)	0.460	0.302–0.700	<0.001
ALI				
<70.06	592 (65.9)		1.000	
≥70.06	307 (34.1)	0.271	0.148–0.496	<0.001
PLR				
<143.73	641 (71.3)		1.000	
≥143.73	258 (28.7)	2.062	1.378–3.084	<0.001
PIV				
<156.18	426 (47.4)		1.000	
≥156.18	473 (52.6)	2.151	1.398–3.311	<0.001
SII				
<487.10	611 (68.0)		1.000	
≥487.10	288 (32.0)	2.511	1.685–3.742	<0.001

FPR, fibrinogen-to-prealbumin ratio; CAR, C-reactive protein-to-albumin ratio; AAPR, albumin-to-alkaline phosphatase ratio; FAR, fibrinogen-to-albumin ratio; APR, alkaline phosphatase-to-prealbumin ratio; RAR, red cell distribution width-to-albumin ratio; AGR.

albumin-to-globulin ratio; NLR, neutrophil-to-lymphocyte ratio; LMR, lymphocyte-to-monocyte ratio; FFA/Alb, free fatty acid-to-albumin ratio; PNI, prognostic nutritional index; ALI, advanced lung cancer inflammation index; PLR, platelet-to-lymphocyte ratio; PIV, pan-immune-inflammation value; SII, systemic immune-inflammation index.

The patients were categorized into two groups based on their survival outcomes at the conclusion of the follow-up period: a survival group (*n* = 802) and a non-survival group (*n* = 97). The purpose was to compare the survival rates of each inflammatory and nutritional composite index between these two groups. The results of the statistical analysis revealed significant differences (*p* < 0.05) among the 15 inflammatory and nutritional complex indices, as presented in **Supplementary Table S3**.

### Comparison of the prognostic power of the inflammatory and nutritional indicators

A C-index analysis was performed on the 15 inflammatory and nutritional indicators to compare their prognostic ability. Compared with other inflammation- and nutrition-based measures, FAR showed the highest C-index of OS in patients with NSCLC at 1, 2, and 3 years: 0.701 (95%CI = 0.681–0.721), 0.667 (95%CI = 0.647–0.687), and 0.639 (95%CI = 0.619–0.659), respectively (**Table 3**). According to the 3-year C-index, FAR, SII, and AAPR were the top 3 inflammation-based indicators. **Supplementary Table S4** shows that FAR, SII, and AAPR contributed significantly to the prognostic value of the TNM classification system.

**Table 3:** C-index of the 15 indicators of overall survival (OS) in non-small cell lung cancer (NSCLC) patients.

Category	C-index		
	1 year	2 years	3 years
FPR	0.663 (0.645–0.681)	0.616 (0.598–0.634)	0.593 (0.575–0.611)
CAR	0.590 (0.570–0.610)	0.598 (0.578–0.618)	0.584 (0.564–0.604)
AAPR	0.672 (0.652–0.692)	0.620 (0.600–0.640)	0.617 (0.597–0.637)
FAR	0.701 (0.681–0.721)	0.667 (0.647–0.687)	0.639 (0.619–0.659)
APR	0.618 (0.598–0.638)	0.592 (0.572–0.612)	0.573 (0.553–0.593)
RAR	0.588 (0.570–0.606)	0.589 (0.571–0.607)	0.570 (0.552–0.588)
AGR	0.520 (0.500–0.540)	0.540 (0.520–0.560)	0.559 (0.539–0.577)
NLR	0.612 (0.592–0.632)	0.609 (0.589–0.629)	0.599 (0.579–0.619)
LMR	0.618 (0.598–0.638)	0.585 (0.565–0.605)	0.599 (0.579–0.619)
FFA/Alb	0.637 (0.617–0.657)	0.561 (0.541–0.581)	0.584 (0.564–0.604)
PNI	0.610 (0.594–0.626)	0.596 (0.580–0.612)	0.586 (0.570–0.602)
ALI	0.641 (0.623–0.659)	0.627 (0.607–0.647)	0.609 (0.589–0.629)
PLR	0.576 (0.558–0.594)	0.574 (0.556–0.592)	0.602 (0.584–0.620)
PIV	0.645 (0.625–0.665)	0.598 (0.578–0.618)	0.597 (0.577–0.617)
SII	0.655 (0.637–0.673)	0.598 (0.580–0.616)	0.622 (0.604–0.640)

FPR, fibrinogen-to-prealbumin ratio; CAR, C-reactive protein-to-albumin ratio; AAPR, albumin-to-alkaline phosphatase ratio; FAR, fibrinogen-to-albumin ratio; APR, alkaline phosphatase-to-prealbumin ratio; RAR, red cell distribution width-to-albumin ratio; AGR.

albumin-to-globulin ratio; NLR, neutrophil-to-lymphocyte ratio; LMR, lymphocyte-to-monocyte ratio; FFA/Alb, free fatty acid-to-albumin ratio; PNI, prognostic nutritional index; ALI, advanced lung cancer inflammation index; PLR, platelet-to-lymphocyte ratio; PIV, pan-immune-inflammation value; SII, systemic immune-inflammation index.

In the different subgroups, FAR had the highest C-index compared with the other indicators in men, non-smokers, adenocarcinomas, and stage II patients (**Supplementary Tables S5**–**S9**). The C-index of PLR was the highest in female patients (**Supplementary Table S5**). SII was the highest in smokers (**Supplementary Table S6**), in those aged <65 and ≥65 years (**Supplementary Table S7**), and in stage III patients (**Supplementary Table S9**). The C-index of AAPR was the highest in non-adenocarcinomas (**Supplementary Table S8**). The C-index of PIV was the highest in stage I patients (**Supplementary Table S9**).

### Association of FAR, SII, and AAPR with clinical outcomes

Overall, FAR, SII, and AAPR were the top 3 inflammatory and nutritional markers that predicted prognosis in patients with NSCLC. **Supplementary Tables S10**–**S12** displays the baseline characteristics of patients with NSCLC stratified by high/low FAR, SII, and AAPR. The Kaplan–Meier curves of FAR, SII, and AAPR for the subgroups of NSCLC patients were constructed and stratified according to sex, smoking status, and pathological type. The results showed that even within subgroups, patients with high FAR, high SII, and low AAPR still had poor OS performance (**Supplementary Figure S2**).

The univariate analysis showed that sex (*p* = 0.012), smoking status (*p* = 0.002), pathologic type (*p* < 0.001), TNM stage (*p* < 0.001), tumor diameter (*p* < 0.001), endovascular thrombus (*p* < 0.001), perineural invasion (*p* = 0.001), FAR (*p* < 0.001), SII (*p* < 0.001), and AAPR (*p* < 0.001) were predictive of OS. In the multivariate Cox regression analysis, only the TNM stage (*p* < 0.001), perineural invasion (*p* = 0.035), and FAR (*p* = 0.037) were independent prognostic factors for OS in patients with NSCLC (**Table 4**).

**Table 4:** Cox regression analysis of overall survival (OS) in all non-small cell lung cancer (NSCLC) patients.

Category	Univariate analysis		Multivariate analysis	
	HR (95% CI)	*p*	HR (95% CI)	*p*
Age (years)				
<65	1.000			
≥65	1.358 (0.908–2.031)	0.136		
Sex				
Male	1.000		1.000	
Female	0.593 (0.395–0.891)	0.012	0.843 (0.465–1.530)	0.575
Smoking status				
Never smoked	1.000		1.000	
Current/past smoking	1.885 (1.264–2.812)	0.002	0.929 (0.533–1.618)	0.795
BMI (kg/m^2^)				
<18.5	1.000			
18.5–23.9	2.324 (0.321–16.826)	0.404		
>24	3.135 (0.432–22.765)	0.259		
ECOG PS score				
0–1	1.000			
≥2	1.195 (0.730–1.955)	0.479		
Respiratory diseases				
No	1.000			
Yes	1.401 (0.910–2.157)	0.126		
Pathologic type				
Adenocarcinoma	1.000		1.000	
Non-adenocarcinoma	2.362 (1.478–3.773)	<0.001	1.041 (0.625–1.733)	0.877
TNM stage				
Stage I	1.000		1.000	
Stage II	106.771 (46.118–247.190)	<0.001	105.558 (43.866–254.011)	<0.001
Stage III	31.486 (13.033–76.066)	<0.001	28.004 (11.263–69.629)	<0.001
Tumor diameter (cm)				
<2.25	1.000		1.000	
≥2.25	4.524 (2.989–6.849)	<0.001	0.926 (0.589–1.455)	0.738
Vascular invasion				
No	1.000			
Yes	1.293 (0.849–1.970)	0.231		
Endovascular thrombus				
No	1.000		1.000	
Yes	3.691 (2.389–5.702)	<0.001	0.717 (0.441–1.165)	0.179
Lymphatic vessel invasion				
No	1.000			
Yes	1.283 (0.848–1.940)	0.239		
Perineural invasion				
No	1.000		1.000	
Yes	3.559 (1.725–7.342)	0.001	2.371 (1.064–5.286)	0.035
FAR				
<0.079	1.000			
≥0.079	2.545 (1.683–3.848)	<0.001	1.601 (1.028–2.495)	0.037
SII				
<487.10	1.000			
≥487.10	2.511 (1.685–3.742)	<0.001	0.770 (0.487–1.217)	0.263
AAPR				
<0.045	1.000			
≥0.045	0.425 (0.280–0.644)	<0.001	1.043 (0.643–1.694)	0.863

FAR, fibrinogen-to-albumin ratio; SII, systemic immune-inflammation index; AAPR, albumin-to-alkaline phosphatase ratio.

Considering that there may be significant differences in the nutritional status and the inflammatory status between patients with early-stage and middle-advanced lung cancer, Cox regression analysis was performed for patients with stage I–II and stage III NSCLC in order to further evaluate the potential prognostic value of FAR, SII, and AAPR in patients with different stages of the disease. The results of the univariate Cox regression analysis are shown in **Supplementary Tables S13**, **S14**. The multivariate Cox regression analysis showed that tumor diameter (*p* = 0.047) and FAR (*p* = 0.049) were independent prognostic factors for OS in patients with stage I–II NSCLC. In those with stage III NSCLC, variables with *p* < 0.1 (SII and perineural invasion) were added to the multifactorial model after correction. However, only perineural invasion (*p* = 0.032) was a strong predictor of the outcome.

## Discussion

Increasing research suggests that inflammation- and nutrition-based indicators are dependable predictors of OS in patients with cancer. Chronic inflammation produces several cytokines that promote the initiation and progression of malignancies through pathophysiological mechanisms (25). Furthermore, malnutrition in patients with cancer may result in a weakened immune function and an increased inflammatory response (26). Cancer patients with lower nutritional markers or higher inflammatory markers tend to have worse outcomes (27). However, the optimal indicator for patients with NSCLC is unclear. Our study evaluated and compared 15 inflammation- and nutrition-based measures and found that FAR had stable and good predictive performance in predicting the prognosis of patients with NSCLC and their subgroups in risk stratification.

Consistent with previous studies (9–20), our study found that NLR, PLR, LMR, SII, PIV, FAR, FPR, AGR, ALI, PNI, AAPR, CAR, RAR, APR, and FFA/Alb were all associated with OS in the univariate analysis. Even RAR, APR, and FFA/Alb, which were not evaluated in previous lung cancer association studies, were found to be independent predictors of prognosis in patients with NSCLC. Some studies have claimed that FAR, SII, and AAPR are associated with OS in patients with NSCLC (12, 19, 28). However, no previous studies have compared these three indicators in these patients. A recent study found that ALI was more effective in predicting the prognosis of patients with lung cancer compared with other inflammatory or nutritional markers (29). Among the 15 indicators related to inflammation and nutrition investigated in this study, FAR had the most accurate predictive ability in assessing the prognosis of patients with NSCLC, surpassing ALI in predictive effectiveness. FAR is an objective, easy-to-use, and simplified approach that helps facilitate the timely, individualized treatment of patients with NSCLC in clinical practice. However, the results need to be confirmed in more prospective studies.

In this study, we also observed that TNM staging combined with FAR, SII, and AAPR had better predictive value than the TNM staging system alone. Subsequently, Kaplan–Meier survival analysis was performed to examine these three indicators. The results showed that higher levels of FAR and SII and lower levels of AAPR were linked to worse OS. However, after excluding potential confounders based on multifactor regression analysis, only FAR was found to be an independent prognostic factor for patients with NSCLC. This suggests that timely intervention should be carried out during the treatment of patients to improve their nutritional status, inhibit inflammatory responses, and improve coagulation function.

The C-index can be used to assess the differentiation ability of various models; that is, all of the research content in the research data are randomly paired. In this cohort, if a patient with a longer survival time is predicted to live longer than another patient with a relatively shorter survival time, the prediction is said to match the actual outcome. In other words, the patient who was predicted to have a high survival rate actually achieved a higher survival rate than the other patient, a phenomenon known as consensus prediction. In other words, the area under the curve (AUC) mainly reflects the predictive power of the model, but the C-index can evaluate the accuracy of the prediction results of various models, which can be simply understood as the C-index is an extension of the AUC, while the AUC is a special case of the C-index. Although the survival analysis and the C-index results all suggested that FAR, SII, and AAPR had a certain predictive value for OS in NSCLC patients, the multivariate Cox regression analysis suggested that only FAR was an independent prognostic factor for OS in NSCLC patients, while SII and AAPR were not. At the same time, in this study, we have observed that the C-index of FAR was better than that of SII and AAPR. These results suggest that FAR is better than SII and AAPR in terms of prognostic value for patients with NSCLC.

As a composite indicator based on fibrinogen and albumin, FAR has been reported to be a potential predictor of adverse outcomes in various malignancies, such as esophageal squamous cell carcinoma (30), hepatocellular carcinoma (31), and NSCLC (28). The mechanism of action of FAR in cancer prognosis can be explained by studying the functions of its components. Research has indicated that fibrinogen levels will increase to different degrees when the body is in a pathophysiological condition, such as a tumor, surgery, infection, inflammation, or trauma (32). Research has also indicated that fibrinogen serves as a cytoskeleton within the tumor extracellular matrix, shielding tumor cells from immune cell attack, facilitating interactions between platelets and circulating tumor cells (CTCs), enhancing platelet adhesion to CTCs, and boosting the metastatic potential of tumor cells (33, 34). Furthermore, fibrinogen can bind directly to the intercellular adhesion molecule-1 (ICAM-1) on endothelial cells, enhancing tumor cell adhesion, proliferation, and migration (35).

Previous studies have shown that nutritional status plays an important prognostic role in disease progression and long-term survival in patients with cancer (36). Albumin is the most abundant circulating protein in plasma and not only reflects the nutritional status of the human body but also participates in the systemic inflammatory response (37). Low albumin levels or poor nutritional status could lead to impaired immune function in tumor patients and promote tumor proliferation, invasion, and migration (38). For the aforementioned reasons, the composite index composed of fibrinogen and albumin is more beneficial for evaluating a patient’s overall condition, and its predictive value is higher than that of single inflammation indicators, such as NLR and PLR.

The study has several limitations. First, this is a retrospective analysis with a single-center design and a limited sample size, which inevitably leads to selection bias in the study subjects and the clinical data collection. Second, there is no clear consensus on the optimal cutoff values for FAR, SII, and AAPR, and the impact of dynamic changes in FAR, SII, and AAPR on long-term prognosis remains to be evaluated. Third, this study is a retrospective analysis with a long follow-up duration, and the recommended NSCLC treatment regimen has been constantly updated. Therefore, prospective, large-sample, multicenter studies are needed in the future to further validate the findings of this study, and subgroup analysis should be conducted using the treatment approach as a stratification factor to explore other variables that are meaningful for prognostic assessment in order to further improve the predictive efficacy of this model.

## Conclusion

In conclusion, among the other indicators, FAR was the best predictor of prognosis in patients with NSCLC. Assessment of FAR can identify patients at potential risk of poor prognosis and is expected to be a useful prognostic marker in clinical practice.

## Data availability statement

The raw data supporting the conclusions of this article will be made available by the authors, without undue reservation.

## Ethics statement

This study was reviewed and approved by Institutional Review Board of Qingdao Municipal Hospital, which waived the informed consent requirement due to the retrospective design of the study. This study complied with the Declaration of Helsinki. The studies were conducted in accordance with the local legislation and institutional requirements. Written informed consent for participation was not required from the participants or the participants’ legal guardians/next of kin in accordance with the national legislation and institutional requirements.

## Author contributions

SM: Formal analysis, Investigation, Methodology, Software, Validation, Visualization, Writing – original draft, Writing – review & editing. LW: Data curation, Methodology, Project administration, Supervision, Visualization, Writing – review & editing.
