# Supplementary material for: Fibrinogen-to-albumin ratio (FAR) is the best biomarker for the overall survival of patients with non-small-cell lung cancer
Source: Front Oncol. 2024 Jun 24;14:1396843. doi: 10.3389/fonc.2024.1396843 (PMC11228243; doi:10.3389/fonc.2024.1396843)
Supplement: Supplementary file 1 [file DataSheet_1.docx]

**Table S1.** Calculation methods of combination in each nutrition/inflammation-based indicator.

| Indicators | Definition or calculation formula |
| --- | --- |
| FPR | Fibrinogen (g/ L)/ prealbumin (mg/L) |
| CAR | C-reactive protein (mg/ L)/ albumin (g/ L) |
| AAPR | albumin (g/ dl)/ [Alkaline Phosphatase](https://www.baidu.com/s?tn=84053098_3_dg&wd=Alkaline%20Phosphatase&usm=1&ie=utf-8&rsv_pq=9b7933410030469f&oq=%E7%A2%B1%E6%80%A7%E7%A3%B7%E9%85%B8%E9%85%B6%E8%8B%B1%E6%96%87%E5%85%A8%E7%A7%B0&rsv_t=eaabgu8ygYZwiMfQ5NC0o1Kwz58DDvWAL6zxbWE3t8lVrGgoESbeTcXZnl7Lde5a%2BiDacw&sa=re_dqa_zy&icon=1) **(IU/L)** |
| FAR | Fibrinogen (g/ L)/ albumin (g/ L) |
| APR | [Alkaline Phosphatase](https://www.baidu.com/s?tn=84053098_3_dg&wd=Alkaline%20Phosphatase&usm=1&ie=utf-8&rsv_pq=9b7933410030469f&oq=%E7%A2%B1%E6%80%A7%E7%A3%B7%E9%85%B8%E9%85%B6%E8%8B%B1%E6%96%87%E5%85%A8%E7%A7%B0&rsv_t=eaabgu8ygYZwiMfQ5NC0o1Kwz58DDvWAL6zxbWE3t8lVrGgoESbeTcXZnl7Lde5a%2BiDacw&sa=re_dqa_zy&icon=1) **(IU/L)/** prealbumin (mg/L) |
| RAR | Red Cell Distribution Width (fl)/ albumin (g/ L) |
| AGR | albumin (g/L)/globulin (g/L) |
| NLR | neutrophil count (×10^9^)/ lymphocyte count (×10^9^) |
| LMR | lymphocyte count (×10^9^)/ monocytes (×10^9^) |
| FFA/Alb | Free fatty acid (**mol/L**)/ albumin (g/ L) |
| PNI | albumin (g/L) + 5×lymphocyte count (×10^9^) |
| ALI | BMI (kg/m^2^) × albumin(g/dl)/NLR (×10^9^) |
| PLR | platelet count (×10^9^)/ lymphocyte count (×10^9^) |
| PIV | neutrophil count (×10^9^) ×platelet count (×10^9^) ×monocytes (×10^9^)/ lymphocyte count (×10^9^) |
| SII | platelet count (×10^9^) × neutrophil count (×10^9^)/lymphocyte count (×10^9^) |

**Table S2.** Specificity and sensitivity of ROC curves for 15 inflammatory and nutritional complex indicators.

| Indicators | AUC | 95% CI | Sensitivity | Specificity |
| --- | --- | --- | --- | --- |
| FPR | 0.628 | 0.568～0.688 | 0.479 | 0.719 |
| CAR | 0.597 | 0.536～0.658 | 0.760 | 0.453 |
| AAPR | 0.357 | 0.298～0.418 | 0.646 | 0.618 |
| FAR | 0.660 | 0.603～0.716 | 0.635 | 0.613 |
| APR | 0.615 | 0.554～0.675 | 0.573 | 0.630 |
| RAR | 0.570 | 0.512～0.628 | 0.448 | 0.688 |
| AGR | 0.403 | 0.343～0.464 | 0.729 | 0.429 |
| NLR | 0.621 | 0.565～0.678 | 0.740 | 0.498 |
| LMR | 0.448 | 0.386～0.511 | 0.552 | 0.559 |
| FFA/Alb | 0.573 | 0.508～0.638 | 0.680 | 0.545 |
| PNI | 0.433 | 0.370～0.496 | 0.354 | 0.796 |
| ALI | 0.374 | 0.319～0.428 | 0.875 | 0.368 |
| PLR | 0.570 | 0.507～0.633 | 0.427 | 0.731 |
| PIV | 0.606 | 0.549～0.662 | 0.688 | 0.494 |
| SII | 0.628 | 0.572～0.684 | 0.521 | 0.704 |

FPR, fibrinogen to prealbumin ratio; CAR, C-reactive protein to albumin ratio; AAPR, albumin to alkaline phosphatase ratio; FAR, fibrinogen to albumin ratio; APR, alkaline phosphatase to prealbumin ratio; RAR, red cell distribution width to albumin ratio; AGR, albumin globulin ratio; NLR, neutrophil to lymphocyte ratio; LMR, lymphocyte to monocyte ratio; FFA/Alb, Free fatty acid to albumin ratio; PNI, prognostic nutritional index; ALI, advanced lung cancer inflammatory index; PLR, platelet to lymphocyte ratio; PIV, Pan-Immune-Inflammation Value; SII, systemic immune inflammation index.

**Table S3.** Survival differences in 15 inflammatory and nutritional complex markers between survival and non-survival groups.

| Category | Survival (n=802) | Non-survival (n=97) | *P - value* |
| --- | --- | --- | --- |
| FPR |  |  |  |
| <0.013 | 576 (71.8) | 51 (52.6) | <0.001 |
| ≥0.013 | 226 (28.2) | 46 (47.4) |  |
| CAR |  |  |  |
| <0.017 | 363 (45.3) | 23 (23.7) | <0.001 |
| ≥0.017 | 439 (54.7) | 74 (76.3) |  |
| AAPR |  |  |  |
| <0.045 | 306 (38.2) | 62 (63.9) | <0.001 |
| ≥0.045 | 496 (61.8) | 35 (36.1) |  |
| FAR |  |  |  |
| <0.079 | 491 (61.2) | 36 (37.1) | <0.001 |
| ≥0.079 | 311 (38.8) | 61 (62.9) |  |
| APR |  |  |  |
| <0.34 | 505 (63.0) | 42 (43.3) | <0.001 |
| ≥0.34 | 297 (37.0) | 55 (56.7) |  |
| RAR |  |  |  |
| <1.13 | 552 (68.8) | 54 (55.7) | 0.011 |
| ≥1.13 | 250 (31.2) | 43 (44.3) |  |
| AGR |  |  |  |
| <1.49 | 458 (57.1) | 70 (72.2) | 0.004 |
| ≥1.49 | 344 (42.9) | 27 (27.8) |  |
| NLR |  |  |  |
| <1.64 | 399 (49.8) | 25 (25.8) | <0.001 |
| ≥1.64 | 403 (50.2) | 72 (74.2) |  |
| LMR |  |  |  |
| <4.17 | 354 (44.1) | 54 (55.7) | 0.040 |
| ≥4.17 | 448 (55.9) | 43 (44.3) |  |
| FFA/Alb |  |  |  |
| <9.81 | 365 (45.5) | 66 (68.0) | <0.001 |
| ≥9.81 | 437 (54.5) | 31 (32.0) |  |
| PNI |  |  |  |
| <45.51 | 164 (20.4) | 34 (35.1) | 0.002 |
| ≥45.51 | 638 (79.6) | 63 (64.9) |  |
| ALI |  |  |  |
| <70.06 | 507 (63.2) | 85 (87.6) | <0.001 |
| ≥70.06 | 295 (36.8) | 12 (12.4) |  |
| PLR |  |  |  |
| <143.73 | 586 (73.1) | 55 (56.7) | 0.001 |
| ≥143.73 | 216 (26.9) | 42 (43.3) |  |
| PIV |  |  |  |
| <156.18 | 396 (49.4) | 30 (30.9) | 0.001 |
| ≥156.18 | 406 (50.6) | 67 (69.1) |  |
| SII |  |  |  |
| <487.10 | 565 (70.4) | 46 (47.4) | <0.001 |
| ≥487.10 | 237 (29.6) | 51 (52.6) |  |

FPR, fibrinogen to prealbumin ratio; CAR, C-reactive protein to albumin ratio; AAPR, albumin to alkaline phosphatase ratio; FAR, fibrinogen to albumin ratio; APR, alkaline phosphatase to prealbumin ratio; RAR, red cell distribution width to albumin ratio; AGR, albumin globulin ratio; NLR, neutrophil to lymphocyte ratio; LMR, lymphocyte to monocyte ratio; FFA/Alb, Free fatty acid to albumin ratio; PNI, prognostic nutritional index; ALI, advanced lung cancer inflammatory index; PLR, platelet to lymphocyte ratio; PIV, Pan-Immune-Inflammation Value; SII, systemic immune inflammation index.

**Table S4.** The C-index of different prognostic models on 899 NSCLC patients.

| Indicators | C-index |
| --- | --- |
| TNM | 0.925 (0.907,0.943) |
| TNM+FAR | 0.933 (0.915,0.951) |
| TNM+SII | 0.927 (0.909,0.945) |
| TNM+AAPR | 0.931 (0.913,0.949) |

FAR, fibrinogen to albumin ratio; SII, systemic immune inflammation index; AAPR, albumin to alkaline phosphatase ratio.

**Table S5.** The C-index of fifteen indicators for OS in patients with NSCLC stratified by Gender.

| Indicators | C-index (95%CI) | |
| --- | --- | --- |
|  | Male | Female |
| FPR | 0.632 (0.607,0.657) | 0.530 (0.506,0.554) |
| CAR | 0.571 (0.542,0.600) | 0.609 (0.582,0.636) |
| AAPR | 0.626 (0.597,0.655) | 0.592 (0.567,0.617) |
| FAR | 0.656 (0.627,0.685) | 0.585 (0.560,0.610) |
| APR | 0.598 (0.571,0.625) | 0.565 (0.538,0.592) |
| RAR | 0.547 (0.518,0.576) | 0.580 (0.569,0.604) |
| AGR | 0.605 (0.576,0.634) | 0.520 (0.493,0.547) |
| NLR | 0.588 (0.559,0.617) | 0.572 (0.547,0.597) |
| LMR | 0.552 (0.523,0.581) | 0.610 (0.585,0.635) |
| FFA/Alb | 0.566 (0.537,0.595) | 0.595 (0.570,0.620) |
| PNI | 0.588 (0.563,0.613) | 0.556 (0.536,0.576) |
| ALI | 0.596 (0.571,0.621) | 0.592 (0.565,0.619) |
| PLR | 0.591 (0.566,0.616) | 0.625 (0.601,0.649) |
| PIV | 0.600 (0.571,0.629) | 0.545 (0.520,0.570) |
| SII | 0.612 (0.583,0.641) | 0.614 (0.592,0.636) |

FPR, fibrinogen to prealbumin ratio; CAR, C-reactive protein to albumin ratio; AAPR, albumin to alkaline phosphatase ratio; FAR, fibrinogen to albumin ratio; APR, alkaline phosphatase to prealbumin ratio; RAR, red cell distribution width to albumin ratio; AGR, albumin globulin ratio; NLR, neutrophil to lymphocyte ratio; LMR, lymphocyte to monocyte ratio; FFA/Alb, Free fatty acid to albumin ratio; PNI, prognostic nutritional index; ALI, advanced lung cancer inflammatory index; PLR, platelet to lymphocyte ratio; PIV, Pan-Immune-Inflammation Value; SII, systemic immune inflammation index.

**Table S6.** The C-index of fifteen indicators for OS in patients with NSCLC stratified by smoking status.

| Indicators | C-index (95%CI) | |
| --- | --- | --- |
|  | Non-smoking | Smoking |
| FPR | 0.598 (0.578,0.618) | 0.580 (0.547,0.613) |
| CAR | 0.633 (0.609,0.657) | 0.530 (0.493,0.567) |
| AAPR | 0.596 (0.572,0.620) | 0.619 (0.584,0.654) |
| FAR | 0.646 (0.624,0.668) | 0.597 (0.560,0.634) |
| APR | 0.578 (0.554,0.602) | 0.582 (0.547,0.617) |
| RAR | 0.595 (0.575,0.615) | 0.508 (0.471,0.545) |
| AGR | 0.556 (0.532,0.580) | 0.578 (0.541,0.615) |
| NLR | 0.590 (0.566,0.614) | 0.580 (0.545,0.615) |
| LMR | 0.611 (0.587,0.635) | 0.540 (0.503,0.577) |
| FFA/Alb | 0.579 (0.555,0.603) | 0.579 (0.546,0.612) |
| PNI | 0.583 (0.565,0.601) | 0.563 (0.532,0.594) |
| ALI | 0.594 (0.570,0.618) | 0.593 (0.564,0.622) |
| PLR | 0.628 (0.606,0.650) | 0.575 (0.544,0.606) |
| PIV | 0.599 (0.575,0.623) | 0.558 (0.523,0.593) |
| SII | 0.595 (0.573,0.617) | 0.628 (0.593,0.663) |

FPR, fibrinogen to prealbumin ratio; CAR, C-reactive protein to albumin ratio; AAPR, albumin to alkaline phosphatase ratio; FAR, fibrinogen to albumin ratio; APR, alkaline phosphatase to prealbumin ratio; RAR, red cell distribution width to albumin ratio; AGR, albumin globulin ratio; NLR, neutrophil to lymphocyte ratio; LMR, lymphocyte to monocyte ratio; FFA/Alb, Free fatty acid to albumin ratio; PNI, prognostic nutritional index; ALI, advanced lung cancer inflammatory index; PLR, platelet to lymphocyte ratio; PIV, Pan-Immune-Inflammation Value; SII, systemic immune inflammation index.

**Table S7.** The *C*-index of fifteen indicators for OS in patients with NSCLC stratified by age.

| Indicators | *C*-index (95%CI) | |
| --- | --- | --- |
|  | Age<65 | Age≥65 |
| FPR | 0.636 (0.614,0.658) | 0.531 (0.500,0.562) |
| CAR | 0.595 (0.570,0.620) | 0.573 (0.542,0.604) |
| AAPR | 0.618 (0.594,0.642) | 0.600 (0.567,0.633) |
| FAR | 0.627 (0.603,0.651) | 0.629 (0.600,0.662) |
| APR | 0.589 (0.565,0.613) | 0.550 (0.517,0.583) |
| RAR | 0.567 (0.545,0.589) | 0.559 (0.528,0.590) |
| AGR | 0.543 (0.518,0.568) | 0.574 (0.541,0.607) |
| NLR | 0.622 (0.597,0.647) | 0.569 (0.536,0.602) |
| LMR | 0.638 (0.607,0.663) | 0.535 (0.502,0.568) |
| FFA/Alb | 0.584 (0.559,0.609) | 0.584 (0.553,0.615) |
| PNI | 0.577 (0.559,0.595) | 0.582 (0.555,0.609) |
| ALI | 0.636 (0.611,0.661) | 0.578 (0.549,0.607) |
| PLR | 0.627 (0.605,0.649) | 0.570 (0.541,0.599) |
| PIV | 0.609 (0.584,0.634) | 0.576 (0.543,0.609) |
| SII | 0.641 (0.617,0.665) | 0.601 (0.572,0.630) |

FPR, fibrinogen to prealbumin ratio; CAR, C-reactive protein to albumin ratio; AAPR, albumin to alkaline phosphatase ratio; FAR, fibrinogen to albumin ratio; APR, alkaline phosphatase to prealbumin ratio; RAR, red cell distribution width to albumin ratio; AGR, albumin globulin ratio; NLR, neutrophil to lymphocyte ratio; LMR, lymphocyte to monocyte ratio; FFA/Alb, Free fatty acid to albumin ratio; PNI, prognostic nutritional index; ALI, advanced lung cancer inflammatory index; PLR, platelet to lymphocyte ratio; PIV, Pan-Immune-Inflammation Value; SII, systemic immune inflammation index.

**Table S8.** The C-index of fifteen indicators for OS in patients with NSCLC stratified by pathological type.

| Indicators | C-index (95%CI) | |
| --- | --- | --- |
|  | Non-Adenocarcinoma | Adenocarcinoma |
| FPR | 0.500 (0.437,0.563) | 0.590 (0.572,0.608) |
| CAR | 0.551 (0.496,0.606) | 0.576 (0.554,0.598) |
| AAPR | 0.577 (0.516,0.638) | 0.603 (0.583,0.623) |
| FAR | 0.551 (0.488,0.614) | 0.629 (0.609,0.649) |
| APR | 0.539 (0.474,0.604) | 0.570 (0.550,0.590) |
| RAR | 0.524 (0.461,0.587) | 0.578 (0.558,0.598) |
| AGR | 0.498 (0.437,0.559) | 0.564 (0.542,0.586) |
| NLR | 0.566 (0.513,0.619) | 0.589 (0.567,0.611) |
| LMR | 0.573 (0.510,0.636) | 0.576 (0.554,0.598) |
| FFA/Alb | 0.573 (0.512,0.634) | 0.593 (0.571,0.615) |
| PNI | 0.538 (0.475,0.601) | 0.575 (0.559,0.591) |
| ALI | 0.540 (0.503,0.577) | 0.613 (0.591,0.635) |
| PLR | 0.516 (0.453,0.579) | 0.615 (0.597,0.633) |
| PIV | 0.560 (0.503,0.617) | 0.587 (0.565,0.609) |
| SII | 0.572 (0.509,0.629) | 0.627 (0.607,0.647) |

FPR, fibrinogen to prealbumin ratio; CAR, C-reactive protein to albumin ratio; AAPR, albumin to alkaline phosphatase ratio; FAR, fibrinogen to albumin ratio; APR, alkaline phosphatase to prealbumin ratio; RAR, red cell distribution width to albumin ratio; AGR, albumin globulin ratio; NLR, neutrophil to lymphocyte ratio; LMR, lymphocyte to monocyte ratio; FFA/Alb, Free fatty acid to albumin ratio; PNI, prognostic nutritional index; ALI, advanced lung cancer inflammatory index; PLR, platelet to lymphocyte ratio; PIV, Pan-Immune-Inflammation Value; SII, systemic immune inflammation index.

**Table S9.** The *C*-index of fifteen indicators for OS in patients with NSCLC stratified by TNM stage.

| Indicators | *C*-index (95%CI) | | |
| --- | --- | --- | --- |
|  | I | II | III |
| FPR | 0.638 (0.620,0.656) | 0.493 (0.420,0.566) | 0.523 (0.433,0.613) |
| CAR | 0.460 (0.438,0.482) | 0.574 (0.505,0.643) | 0.529 (0.441,0.617) |
| AAPR | 0.597 (0.577,0.617) | 0.543 (0.470,0.616) | 0.531 (0.437,0.625) |
| FAR | 0.506 (0.486,0.526) | 0.673 (0.600,0.746) | 0.529 (0.439,0.619) |
| APR | 0.426 (0.406,0.446) | 0.524 (0.451,0.597) | 0.506 (0.414,0.598) |
| RAR | 0.460 (0.440,0.480) | 0.546 (0.470,0.622) | 0.546 (0.458,0.634) |
| AGR | 0.563 (0.541,0.585) | 0.529 (0.456,0.602) | 0.515 (0.423,0.607) |
| NLR | 0.555 (0.533,0.577) | 0.557 (0.488,0.626) | 0.548 (0.456,0.640) |
| LMR | 0.522 (0.500,0.544) | 0.557 (0.486,0.628) | 0.490 (0.396,0.584) |
| FFA/Alb | 0.585 (0.563,0.607) | 0.639 (0.566,0.712) | 0.548 (0.454,0.642) |
| PNI | 0.547 (0.531,0.563) | 0.569 (0.495,0.643) | 0.544 (0.460,0.628) |
| ALI | 0.497 (0.475,0.519) | 0.534 (0.477,0.591) | 0.555 (0.475,0.635) |
| PLR | 0.655 (0.637,0.673) | 0.550 (0.481,0.619) | 0.535 (0.443,0.627) |
| PIV | 0.674 (0.652,0.696) | 0.525 (0.454,0.596) | 0.545 (0.455,0.635) |
| SII | 0.571 (0.551,0.591) | 0.563 (0.490,0.636) | 0.558 (0.470,0.646) |

FPR, fibrinogen to prealbumin ratio; CAR, C-reactive protein to albumin ratio; AAPR, albumin to alkaline phosphatase ratio; FAR, fibrinogen to albumin ratio; APR, alkaline phosphatase to prealbumin ratio; RAR, red cell distribution width to albumin ratio; AGR, albumin globulin ratio; NLR, neutrophil to lymphocyte ratio; LMR, lymphocyte to monocyte ratio; FFA/Alb, Free fatty acid to albumin ratio; PNI, prognostic nutritional index; ALI, advanced lung cancer inflammatory index; PLR, platelet to lymphocyte ratio; PIV, Pan-Immune-Inflammation Value; SII, systemic immune inflammation index.

**Table S10.** Baseline characteristics stratified by FAR.

| Baseline | Low FAR (n = 527) | High FAR (n = 372) | *P* |
| --- | --- | --- | --- |
| Age (year), median (IQR) | 60.00 (54.00 - 66.00) | 64.00 (58.25 - 69.00) | <0.001 |
| Gender, n (%) |  |  | 0.222 |
| Male | 233 (44.2) | 180 (48.4) |  |
| Female | 294 (55.8) | 192 (51.6) |  |
| BMI, median (IQR) | 24.54 (22.27 - 26.99) | 24.39 (22.49 - 26.83) | 0.999 |
| Smoking status, n (%) |  |  | 0.001 |
| Never smoking | 384 (72.9) | 232 (62.4) |  |
| Current or former smoker | 143 (27.1) | 140 (37.6) |  |
| Respiratory diseases, n (%) |  |  | 0.001 |
| No | 420 (79.7) | 259 (69.6) |  |
| Yes | 107 (20.3) | 113 (30.4) |  |
| ECOG PS score, n (%) |  |  | 0.012 |
| 0 - 1 | 442 (83.9) | 287 (77.2) |  |
| ≥2 | 85 (16.1) | 85 (22.8) |  |
| Tumor diameter (cm), median (IQR) | 1.40 (1.00 - 2.00) | 1.90 (1.20 - 3.00) | <0.001 |
| Pathologic types, n (%) |  |  | <0.001 |
| Adenocarcinoma | 495 (93.9) | 298 (80.1) |  |
| Non-Adenocarcinoma | 32 (6.1) | 74 (19.9) |  |
| TNM stage, n (%) |  |  | <0.001 |
| I | 453 (86.0) | 254 (68.3) |  |
| II | 35 (6.6) | 52 (14.0) |  |
| III | 39 (7.4) | 66 (17.7) |  |
| Vascular invasion |  |  | 0.327 |
| No | 203 (38.5) | 131 (35.2) |  |
| Yes | 324 (61.5) | 241 (64.8) |  |
| Endovascular thrombus |  |  | <0.001 |
| No | 488 (92.6) | 312 (83.9) |  |
| Yes | 39 (7.4) | 60 (16.1) |  |
| lymphatic vessel invasion |  |  | 0.298 |
| No | 211 (40.0) | 136 (36.6) |  |
| Yes | 316 (60.0) | 236 (63.4) |  |
| Perineural invasion |  |  | 0.037 |
| No | 518 (98.3) | 357 (96.0) |  |
| Yes | 9 (1.7) | 15 (4.0) |  |
| FPR, median (IQR) | 0.010 (0.008 - 0.011) | 0.015 (0.012 - 0.019) | <0.001 |
| CAR, median (IQR) | 0.014 (0.012 - 0.041) | 0.059 (0.017 - 0.210) | <0.001 |
| AAPR, median (IQR) | 0.052 (0.044 - 0.062) | 0.043 (0.036 - 0.051) | <0.001 |
| APR, median (IQR) | 0.272 (0.232 - 0.341) | 0.361 (0.279 - 0.461) | <0.001 |
| RAR, median (IQR) | 1.037 (0.946 - 1.120) | 1.122 (1.024 - 1.246) | <0.001 |
| AGR, median (IQR) | 1.515 (1.376 - 1.654) | 1.331 (1.160 - 1.462) | <0.001 |
| NLR, median (IQR) | 1.582 (1.241 - 2.078) | 1.866 (1.372 - 2.630) | <0.001 |
| LMR, median (IQR) | 4.591 (3.500 - 5.903) | 4.000 (2.982 - 5.252) | <0.001 |
| FFA/Alb, median (IQR) | 9.792 (6.804 - 11.255) | 10.345 (7.355 - 12.121) | 0.003 |
| PNI, median (IQR) | 50.530 (47.470 - 53.840) | 47.460 (44.140 - 51.285) | <0.001 |
| ALI, median (IQR) | 63.455 (47.208 - 84.877) | 49.467 (34.073 - 70.049) | <0.001 |
| PLR, median (IQR) | 117.195 (93.333 - 142.748) | 124.794 (96.230 - 166.724) | 0.002 |
| PIV, median (IQR) | 142.140 (98.759 - 229.979) | 199.347 (128.751 - 357.952) | <0.001 |
| SII, median (IQR) | 351.502 (253.069 - 492.153) | 432.544 (300.223 - 666.849) | <0.001 |

FPR, fibrinogen to prealbumin ratio; CAR, C-reactive protein to albumin ratio; AAPR, albumin to alkaline phosphatase ratio; FAR, fibrinogen to albumin ratio; APR, alkaline phosphatase to prealbumin ratio; RAR, red cell distribution width to albumin ratio; AGR, albumin globulin ratio; NLR, neutrophil to lymphocyte ratio; LMR, lymphocyte to monocyte ratio; FFA/Alb, Free fatty acid to albumin ratio; PNI, prognostic nutritional index; ALI, advanced lung cancer inflammatory index; PLR, platelet to lymphocyte ratio; PIV, Pan-Immune-Inflammation Value; SII, systemic immune inflammation index.

**Table S11.** Baseline characteristics stratified by SII.

| Baseline | Low SII (n = 611) | High SII (n = 288) | *P* |
| --- | --- | --- | --- |
| Age (year), median (IQR) | 62.00 (56.00 - 68.00) | 62.00 (54.00 - 67.00) | 0.286 |
| Gender, n (%) |  |  | <0.001 |
| Male | 253 (41.4) | 160 (55.6) |  |
| Female | 358 (58.6) | 128 (44.4) |  |
| BMI, median (IQR) | 24.58 (22.49 - 27.25) | 24.19 (22.13 - 26.33) | 0.007 |
| Smoking status, n (%) |  |  | <0.001 |
| Never smoking | 443 (72.5) | 173 (60.1) |  |
| Current or former smoker | 168 (27.5) | 115 (39.9) |  |
| Respiratory diseases, n (%) |  |  | 0.030 |
| No | 475 (77.7) | 204 (70.8) |  |
| Yes | 136 (22.3) | 84 (29.2) |  |
| ECOG PS score, n (%) |  |  | 0.649 |
| 0 - 1 | 498 (81.5) | 231 (80.2) |  |
| ≥2 | 113 (18.5) | 57 (19.8) |  |
| Tumor diameter (cm), median (IQR) | 1.50 (1.00 - 2.20) | 1.75 (1.20 - 3.00) | <0.001 |
| Pathologic types, n (%) |  |  | 0.001 |
| Adenocarcinoma | 555 (90.8) | 238 (82.6) |  |
| Non-Adenocarcinoma | 56 (9.2) | 50 (17.4) |  |
| TNM stage, n (%) |  |  | <0.001 |
| I | 508 (83.1) | 199 (69.1) |  |
| II | 51 (8.3) | 36 (12.5) |  |
| III | 52 (8.5) | 53 (18.4) |  |
| Vascular invasion |  |  | 0.416 |
| No | 233 (38.1) | 101 (35.1) |  |
| Yes | 378 (61.9) | 187 (64.9) |  |
| Endovascular thrombus |  |  | 0.006 |
| No | 556 (91.0) | 244 (84.7) |  |
| Yes | 55 (9.0) | 44 (15.3) |  |
| lymphatic vessel invasion |  |  | 0.464 |
| No | 241 (39.4) | 106 (36.8) |  |
| Yes | 370 (60.6) | 182 (63.2) |  |
| Perineural invasion |  |  | 1.000 |
| No | 595 (97.4) | 280 (97.2) |  |
| Yes | 16 (2.6) | 8 (2.8) |  |
| FPR, median (IQR) | 0.011 (0.009 - 0.013) | 0.012 (0.009 - 0.017) | <0.001 |
| CAR, median (IQR) | 0.017 (0.012 - 0.055) | 0.053 (0.016 - 0.284) | <0.001 |
| AAPR, median (IQR) | 0.049 (0.041 - 0.060) | 0.046 (0.037 - 0.055) | <0.001 |
| FAR, median (IQR) | 0.074 (0.064 - 0.085) | 0.083 (0.069 - 0.108) | <0.001 |
| APR, median (IQR) | 0.294 (0.240 - 0.377) | 0.318 (0.256 - 0.449) | <0.001 |
| RAR, median (IQR) | 1.066 (0.981 - 1.151) | 1.080 (0.989 - 1.198) | 0.035 |
| AGR, median (IQR) | 1.463 (1.316 - 1.618) | 1.381 (1.218 - 1.564) | <0.001 |
| NLR, median (IQR) | 1.420 (1.150 - 1.740) | 2.656 (2.142 - 3.669) | <0.001 |
| LMR, median (IQR) | 4.864 (3.895 - 6.120) | 3.217 (2.395 - 4.236) | <0.001 |
| FFA/Alb, median (IQR) | 9.957 (6.761 - 11.417) | 10.031 (7.390 - 12.053) | 0.079 |
| PNI, median (IQR) | 50.290 (46.890 - 53.560) | 47.500 (43.833 - 51.223) | <0.001 |
| ALI, median (IQR) | 70.005 (55.017 - 88.408) | 35.592 (24.559 - 46.026) | <0.001 |
| PLR, median (IQR) | 105.833 (87.097 - 126.347) | 161.169 (135.347 - 198.353) | <0.001 |
| PIV, median (IQR) | 128.669 (89.143 - 167.780) | 353.897 (255.568 - 579.996) | <0.001 |

FPR, fibrinogen to prealbumin ratio; CAR, C-reactive protein to albumin ratio; AAPR, albumin to alkaline phosphatase ratio; FAR, fibrinogen to albumin ratio; APR, alkaline phosphatase to prealbumin ratio; RAR, red cell distribution width to albumin ratio; AGR, albumin globulin ratio; NLR, neutrophil to lymphocyte ratio; LMR, lymphocyte to monocyte ratio; FFA/Alb, Free fatty acid to albumin ratio; PNI, prognostic nutritional index; ALI, advanced lung cancer inflammatory index; PLR, platelet to lymphocyte ratio; PIV, Pan-Immune-Inflammation Value; SII, systemic immune inflammation index.

**Table S12.** Baseline characteristics stratified by AAPR.

| Baseline | Low AAPR (n = 368) | High AAPR (n = 531) | *P* |
| --- | --- | --- | --- |
| Age (year), median (IQR) | 64.00 (58.00 - 69.00) | 61.00 (54.00 - 66.00) | <0.001 |
| Gender, n (%) |  |  | 0.892 |
| Male | 168 (45.7) | 245 (46.1) |  |
| Female | 200 (54.3) | 286 (53.9) |  |
| BMI, median (IQR) | 24.44 (22.41 - 26.63) | 24.52 (22.43 - 27.10) | 0.366 |
| Smoking status, n (%) |  |  | 0.109 |
| Never smoking | 241 (65.5) | 375 (70.6) |  |
| Current or former smoker | 127 (34.5) | 156 (29.4) |  |
| Respiratory diseases, n (%) |  |  | 0.009 |
| No | 261 (70.9) | 418 (78.7) |  |
| Yes | 107 (29.1) | 113 (21.3) |  |
| ECOG PS score, n (%) |  |  | 0.009 |
| 0 - 1 | 283 (76.9) | 446 (84.0) |  |
| ≥2 | 85 (23.1) | 85 (16.0) |  |
| Tumor diameter (cm), median (IQR) | 1.70 (1.13 - 2.70) | 1.50 (1.00 - 2.20) | <0.001 |
| Pathologic types, n (%) |  |  | <0.001 |
| Adenocarcinoma | 305 (82.9) | 488 (91.9) |  |
| Non-Adenocarcinoma | 63 (17.1) | 43 (8.1) |  |
| TNM stage, n (%) |  |  | <0.001 |
| I | 258 (70.1) | 449 (84.6) |  |
| II | 49 (13.3) | 38 (7.2) |  |
| III | 61 (16.6) | 44 (8.3) |  |
| Vascular invasion |  |  | 0.528 |
| No | 132 (35.9) | 202 (38.0) |  |
| Yes | 236 (64.1) | 329 (62.0) |  |
| Endovascular thrombus |  |  | 0.236 |
| No | 322 (87.5) | 478 (90.0) |  |
| Yes | 46 (12.5) | 53 (10.0) |  |
| lymphatic vessel invasion |  |  | 0.781 |
| No | 140 (38.0) | 207 (39.0) |  |
| Yes | 228 (62.0) | 324 (61.0) |  |
| Perineural invasion |  |  | 0.676 |
| No | 357 (97.0) | 518 (97.6) |  |
| Yes | 11 (3.0) | 13 (2.4) |  |
| FPR, median (IQR) | 0.013 (0.010 - 0.016) | 0.010 (0.009 - 0.012) | <0.001 |
| CAR, median (IQR) | 0.038 (0.014 - 0.149) | 0.017 (0.012 - 0.058) | <0.001 |
| FAR, median (IQR) | 0.085 (0.073 - 0.103) | 0.071 (0.061 - 0.081) | <0.001 |
| APR, median (IQR) | 0.403 (0.342 - 0.493) | 0.257 (0.221 - 0.300) | <0.001 |
| RAR, median (IQR) | 1.105 (1.007 - 1.214) | 1.049 (0.957 - 1.137) | <0.001 |
| AGR, median (IQR) | 1.359 (1.188 - 1.502) | 1.494 (1.350 - 1.654) | <0.001 |
| NLR, median (IQR) | 1.814 (1.295 - 2.496) | 1.622 (1.261 - 2.151) | 0.003 |
| LMR, median (IQR) | 4.239 (3.182 - 5.702) | 4.447 (3.349 - 5.688) | 0.317 |
| FFA/Alb, median (IQR) | 10.125 (6.915 - 11.957) | 9.911 (6.963 - 11.334) | 0.171 |
| PNI, median (IQR) | 48.345 (44.445 - 51.938) | 49.980 (47.030 - 53.330) | <0.001 |
| ALI, median (IQR) | 53.057 (36.203 - 73.264) | 61.730 (45.171 - 81.897) | <0.001 |
| PLR, median (IQR) | 126.497 (97.377 - 163.702) | 115.464 (92.963 - 143.716) | 0.003 |
| PIV, median (IQR) | 184.196 (121.049 - 321.721) | 147.489 (102.953 - 240.214) | <0.001 |
| SII, median (IQR) | 420.049 (299.845 - 642.694) | 354.322 (256.041 - 509.385) | <0.001 |

FPR, fibrinogen to prealbumin ratio; CAR, C-reactive protein to albumin ratio; AAPR, albumin to alkaline phosphatase ratio; FAR, fibrinogen to albumin ratio; APR, alkaline phosphatase to prealbumin ratio; RAR, red cell distribution width to albumin ratio; AGR, albumin globulin ratio; NLR, neutrophil to lymphocyte ratio; LMR, lymphocyte to monocyte ratio; FFA/Alb, Free fatty acid to albumin ratio; PNI, prognostic nutritional index; ALI, advanced lung cancer inflammatory index; PLR, platelet to lymphocyte ratio; PIV, Pan-Immune-Inflammation Value; SII, systemic immune inflammation index.

**Table S13.** Cox regression analysis of OS in patients with stage I to II NSCLC.

| **Category** | **Univariate analysis** | |  | **Multivariate analysis** | |
| --- | --- | --- | --- | --- | --- |
|  | ***HR (95% CI)*** | ***P*** |  | ***HR (95% CI)*** | ***P*** |
| **Age (years)** |  |  |  |  |  |
| <65 | 1.000 |  |  | 1.000 |  |
| ≥65 | 1.99 (1.02～3.86) | 0.043 |  | 1.56 (0.79～3.07) | 0.200 |
| **Gender** |  |  |  |  |  |
| Male | 1.000 |  |  |  |  |
| Female | 0.85 (0.43～1.65) | 0.625 |  |  |  |
| **Smoking status** |  |  |  |  |  |
| Never smoked |  |  |  |  |  |
| Current/past smoking | 1.61 (0.82～3.14) | 0.167 |  |  |  |
| **ECOG PS score** |  |  |  |  |  |
| 0 - 1 | 1.000 |  |  |  |  |
| ≥2 | 0.63 (0.22～1.78) | 0.380 |  |  |  |
| **Disease of respiratory system** |  |  |  |  |  |
| No | 1.000 |  |  |  |  |
| Yes | 1.63 (0.81～3.29) | 0.172 |  |  |  |
| **Pathological type** |  |  |  |  |  |
| Adenocarcinoma | 1.000 |  |  | 1.000 |  |
| Non-adenocarcinoma | 2.63 (1.23～5.63) | 0.013 |  | 1.30 (0.57～2.95) | 0.535 |
| **Tumor diameter (cm)** |  |  |  |  |  |
| <2.25 | 1.000 |  |  | 1.000 |  |
| ≥2.25 | 2.94 (1.51～5.71) | 0.001 |  | 2.03 (1.01～4.08) | 0.047 |
| **Vascular invasion** |  |  |  |  |  |
| No | 1.000 |  |  |  |  |
| Yes | 0.60 (0.31～1.17) | 0.137 |  |  |  |
| **Endovascular thrombus** |  |  |  |  |  |
| No | 1.000 |  |  | 1.000 |  |
| Yes | 2.78 (1.21～6.38) | 0.016 |  | 1.66 (0.70～3.97) | 0.252 |
| **lymphatic vessel invasion** |  |  |  |  |  |
| No | 1.000 |  |  |  |  |
| Yes | 0.61 (0.31～1.2) | 0.153 |  |  |  |
| **Perineural invasion** |  |  |  |  |  |
| No | 1.000 |  |  |  |  |
| Yes | 2.22 (0.53～9.26) | 0.276 |  |  |  |
| **FAR** |  |  |  |  |  |
| <0.079 | 1.000 |  |  | 1.000 |  |
| ≥0.079 | 2.97 (1.49～5.94) | 0.002 |  | 2.09 (1.00～4.36) | 0.049 |
| **SII** |  |  |  |  |  |
| <487.10 | 1.000 |  |  |  |  |
| ≥487.10 | 1.63 (0.83～3.2) | 0.158 |  |  |  |
| **AAPR** |  |  |  |  |  |
| <0.045 | 1.000 |  |  | 1.000 |  |
| ≥0.045 | 0.44 (0.22～0.88) | 0.021 |  | 0.65 (0.31～1.36) | 0.256 |

FAR, fibrinogen to albumin ratio; SII, systemic immune inflammation index; AAPR, albumin to alkaline phosphatase ratio.

**Table S14.** Cox regression analysis of OS in patients with stage III NSCLC.

| **Category** | **Univariate analysis** | |  | **Multivariate analysis** | |
| --- | --- | --- | --- | --- | --- |
|  | ***HR (95% CI)*** | ***P*** |  | ***HR (95% CI)*** | ***P*** |
| **Age (years)** |  |  |  |  |  |
| <65 |  |  |  |  |  |
| ≥65 | 1.08 (0.65～1.81) | 0.762 |  |  |  |
| **Gender** |  |  |  |  |  |
| Male |  |  |  |  |  |
| Female | 0.83 (0.50～1.40) | 0.488 |  |  |  |
| **Smoking status** |  |  |  |  |  |
| Never smoked |  |  |  |  |  |
| Current/past smoking | 0.99 (0.60～1.63) | 0.976 |  |  |  |
| **ECOG PS score** |  |  |  |  |  |
| 0 - 1 |  |  |  |  |  |
| ≥2 | 1.26 (0.71～2.23) | 0.429 |  |  |  |
| **Disease of respiratory system** |  |  |  |  |  |
| No |  |  |  |  |  |
| Yes | 1.28 (0.73～2.23) | 0.386 |  |  |  |
| **Pathological type** |  |  |  |  |  |
| Adenocarcinoma |  |  |  |  |  |
| Non-adenocarcinoma | 1.12 (0.62～2.03) | 0.706 |  |  |  |
| **Tumor diameter (cm)** |  |  |  |  |  |
| <2.25 |  |  |  |  |  |
| ≥2.25 | 1.07 (0.62～1.86) | 0.807 |  |  |  |
| **Vascular invasion** |  |  |  |  |  |
| No |  |  |  |  |  |
| Yes | 1.51 (0.82～2.77) | 0.182 |  |  |  |
| **Endovascular thrombus** |  |  |  |  |  |
| No |  |  |  |  |  |
| Yes | 0.74 (0.44～1.25) | 0.258 |  |  |  |
| **lymphatic vessel invasion** |  |  |  |  |  |
| No |  |  |  |  |  |
| Yes | 1.09 (0.61～1.95) | 0.778 |  |  |  |
| **Perineural invasion** |  |  |  |  |  |
| No |  |  |  |  |  |
| Yes | 2.38 (1.02～5.56) | 0.045 |  | 2.54 (1.08～5.94) | 0.032 |
| **FAR** |  |  |  |  |  |
| <0.079 |  |  |  |  |  |
| ≥0.079 | 1.11 (0.66～1.86) | 0.694 |  |  |  |
| **SII** |  |  |  |  |  |
| <487.10 |  |  |  |  |  |
| ≥487.10 | 1.57 (0.95～2.62) | 0.080 |  | 1.62 (0.97～2.70) | 0.063 |
| **AAPR** |  |  |  |  |  |
| <0.045 |  |  |  |  |  |
| ≥0.045 | 0.76 (0.45～1.27) | 0.299 |  |  |  |

FAR, fibrinogen to albumin ratio; SII, systemic immune inflammation index; AAPR, albumin to alkaline phosphatase ratio.


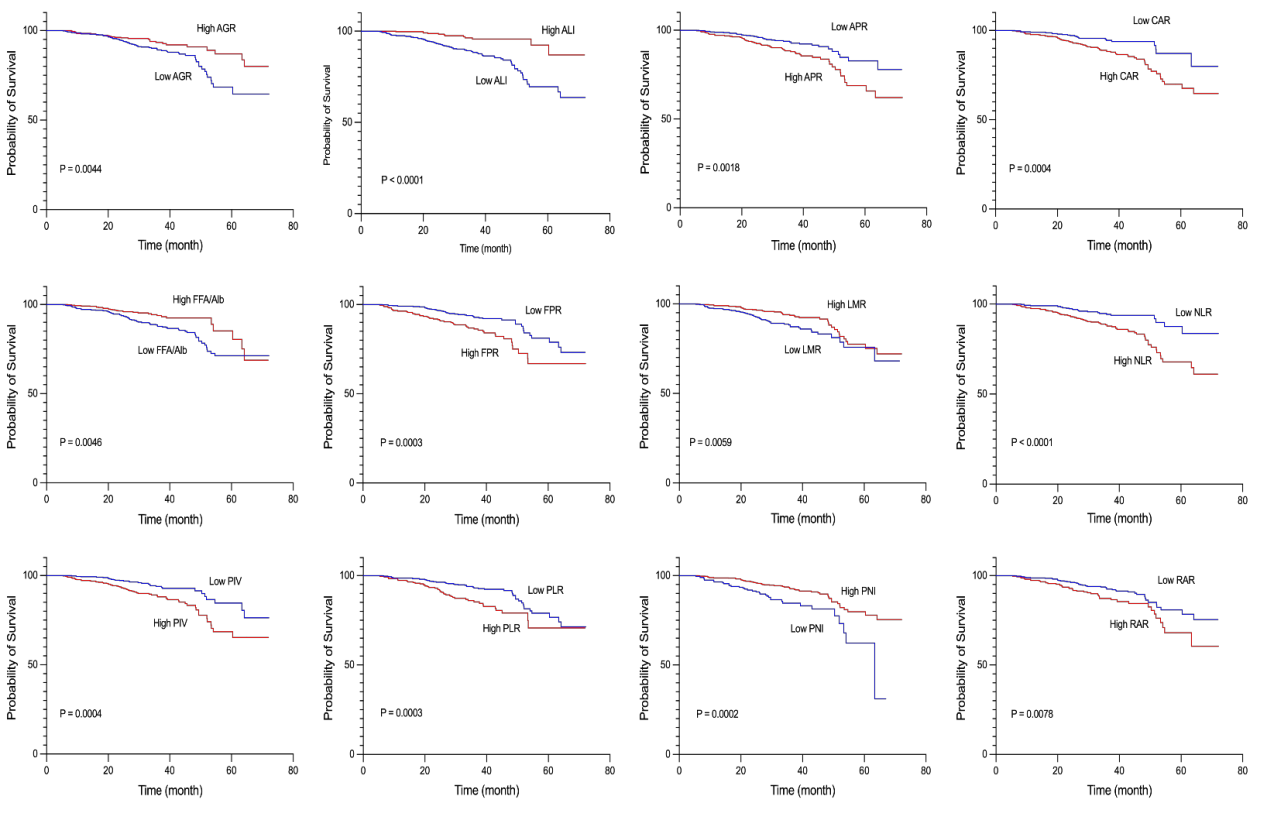


**Figure S1.** The Kaplan-Meier curves of the other twelve indicators in patients with NSCLC.


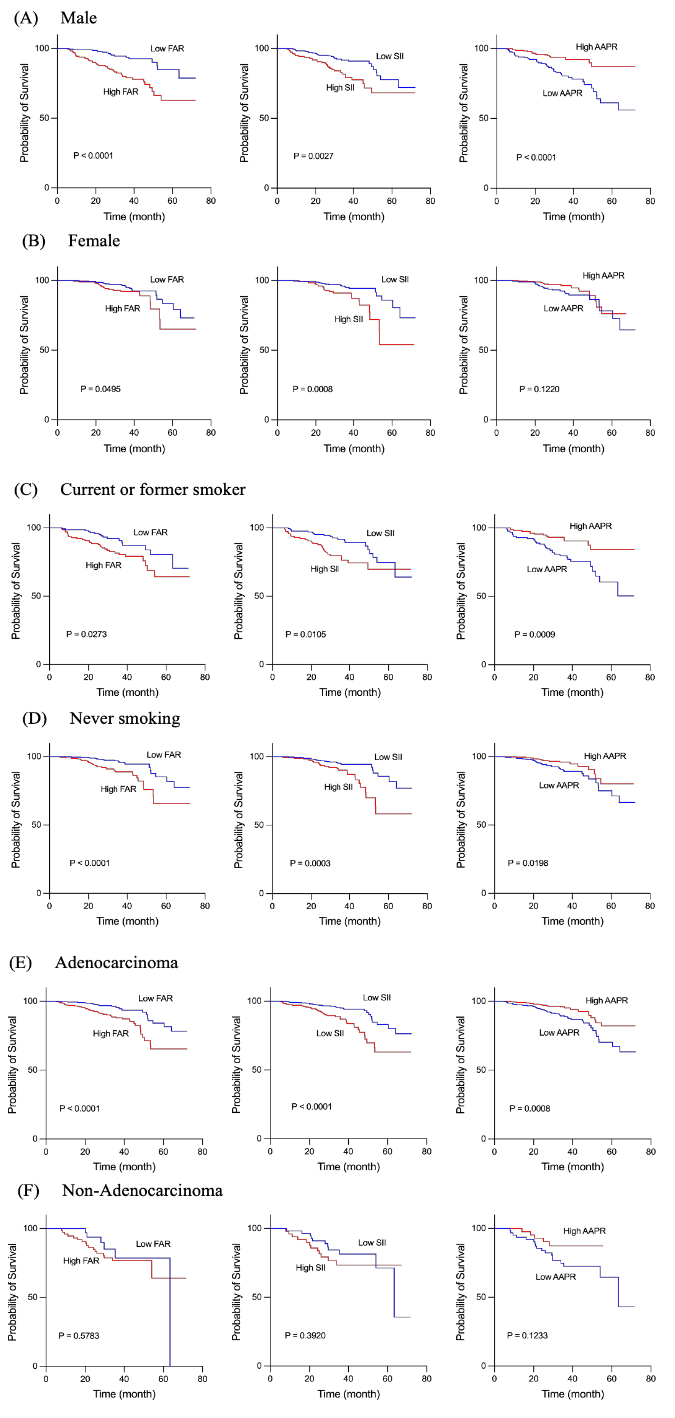


**Figure S2.** Kaplan-Meier curves of the FAR, SII and AAPR in NSCLC patients stratified by gender, smoking status and pathological type: (A) male patients, (B) female patients, (C) smoking; (D) non-smoking; (E) adenocarcinoma; (F) non-adenocarcinoma.
